# poolHiTS: A Shifted Transversal Design based pooling strategy for high-throughput drug screening

**DOI:** 10.1186/1471-2105-9-256

**Published:** 2008-05-30

**Authors:** Raghunandan M Kainkaryam, Peter J Woolf

**Affiliations:** 1 Department of Chemical Engineering, University of Michigan, Ann Arbor, MI, USA; 2 Bioinformatics Program, University of Michigan, Ann Arbor, MI, USA

## Abstract

**Background:**

A key goal of drug discovery is to increase the throughput of small molecule screens without sacrificing screening accuracy. High-throughput screening (HTS) in drug discovery involves testing a large number of compounds in a biological assay to identify active compounds. Normally, molecules from a large compound library are tested individually to identify the activity of each molecule. Usually a small number of compounds are found to be active, however the presence of false positive and negative testing errors suggests that this one-drug one-assay screening strategy can be significantly improved. Pooling designs are testing schemes that test mixtures of compounds in each assay, thereby generating a screen of the whole compound library in fewer tests. By repeatedly testing compounds in different combinations, pooling designs also allow for error-correction. These pooled designs, for specific experiment parameters, can be simply and efficiently created using the Shifted Transversal Design (STD) pooling algorithm. However, drug screening contains a number of key constraints that require specific modifications if this pooling approach is to be useful for practical screen designs.

**Results:**

In this paper, we introduce a pooling strategy called poolHiTS (Pooled High-Throughput Screening) which is based on the STD algorithm. In poolHiTS, we implement a limit on the number of compounds that can be mixed in a single assay. In addition, we show that the STD-based pooling strategy is limited in the error-correction that it can achieve. Due to the mixing constraint, we show that it is more efficient to split a large library into smaller blocks of compounds, which are then tested using an optimized strategy repeated for each block. We package the optimal block selection algorithm into poolHiTS. The MATLAB codes for the poolHiTS algorithm and the corresponding decoding strategy are also provided.

**Conclusion:**

We have produced a practical version of STD algorithm for pooled drug screens. This pooling strategy provides both assay compression and error-correction capabilities that can both accelerate and reduce the overall cost of HTS in drug discovery.

## Background

Advances in automation and miniaturization of experiments and the development of reliable biological assays have made high-throughput screening (HTS) a vital step in the drug discovery process [1-3]. HTS involves the testing of a large number of candidate molecules on a biological target to identify potential drug molecules. Detection technologies such as the scintillation proximity assay (SPA), fluorescence polarization (FP) and fluorescence resonance energy transfer (FRET) are used to identify the target-binding activity of the compounds [4-6]. Screening more than 100,000 compounds a day on a biological target has become routine, but largely through increases in assay automation via liquid handling robots and assay parallelization [7,8]. In these assays, the most common practice is to individually test each molecule against a standardized target. Usually, only a small fraction of a compound library shows activity, while the majority of the compounds show no activity. Unfortunately, because each compound is tested only once, the presence of experimental errors (particularly false negative errors) requires substaintal efforts to validate most HTS results [9]. Due to the large size of these chemical libraries (> 50, 000 compounds) replicate screening is prohibitive. Therefore an arbitrary number of active compounds (hits) are usually chosen for secondary screening to identify inactive compounds that erroneously passed through the primary screen [10]. Disappointingly, active compounds that were missed due to false negative results in the primary assay cannot be identified from this approach and are therefore lost.

One method to reduce the sensitivity of an assay to false postitive and false negative results is pooling. In a pooling design, each compound is tested multiple times in combination with other compounds. Since very few compounds in a library are active in the assay, pooling effectively provides internal replicate measurements to confirm compound activity. However, constructing efficient pooling designs is difficult, as one would ideally like to guarentee correct identification of a known number of active compounds while correcting for random experimental errors. The general problem of pooling designs has been well studied and is described elsewhere [11].

In 2006, a novel pooling design method called shifted transversal design (STD) was introduced for biological assay design [12]. STD is based on the dual objectives of (1) minimizing the number of times any two compounds appear together in a test and (2) maintaining the pool sizes roughly equal. When compared to other pooling designs, STD provides similar or better performance in nearly every area. In this paper, we introduce a pooling strategy called poolHiTS, based on the STD algorithm, specific for the purposes of drug screening. First, we prove a limit to the error-correcting capacity of STD-based pooling strategies. This limit is important for drug screening as it dictates the error limit in an assay that can be used with a pooling design of this kind. Second, we modifiy the pooling design algorithm to limit the number of drugs tested in each assay, thereby enforcing a realistic experimental constraint of HTS. Third, we introduce a block design method that both simplifies and improves the assay design.

## Results and Discussion

### Preliminaries – STD-based pooling strategy

**Figure 1:**
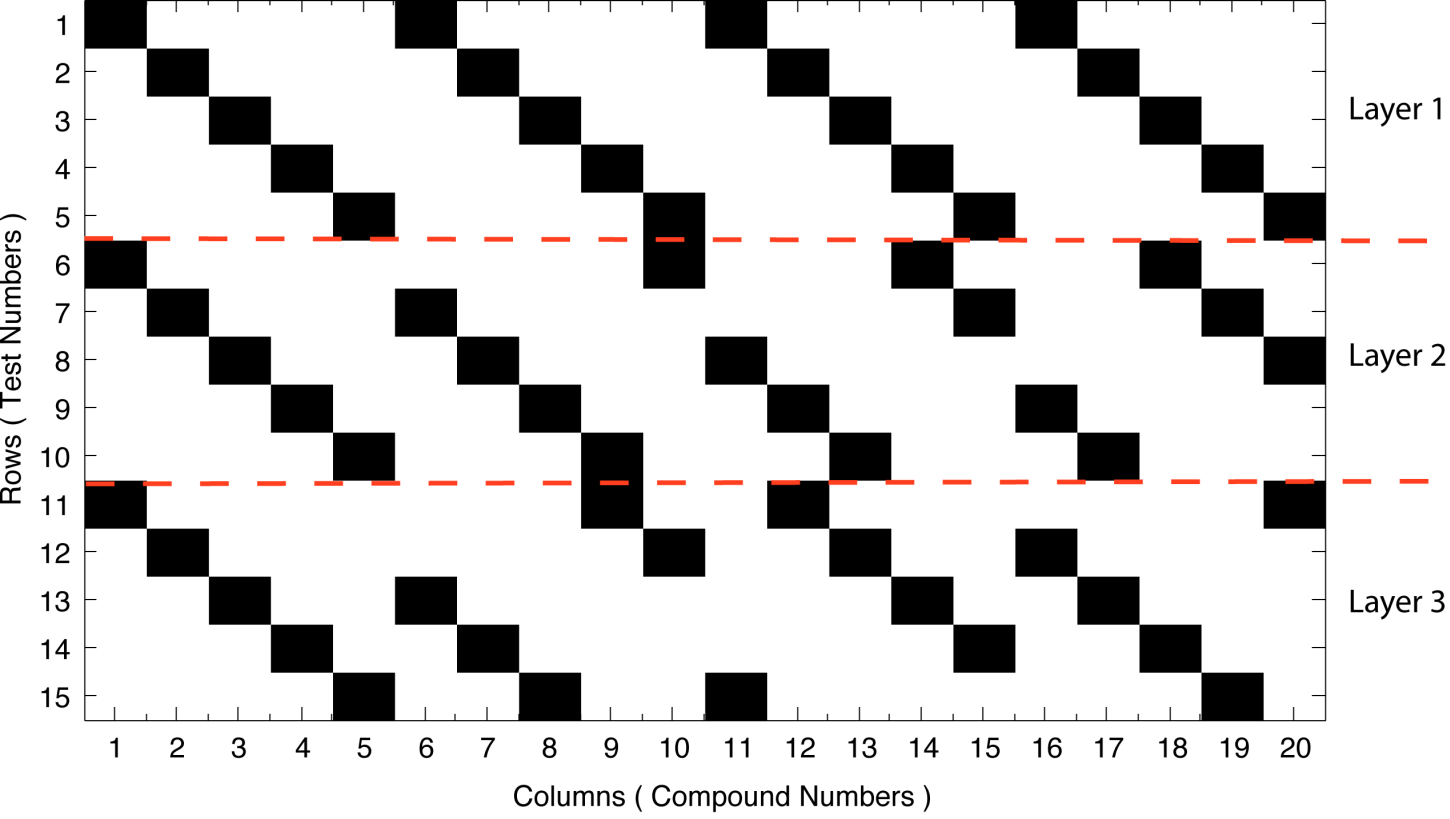
**Example of a STD matrix**. An example of a pooling design for 20 compounds, expecting at most 2 active compounds and 0 errors in testing, requiring a 15 × 20 binary matrix, created using STD(*n *= 20; *q *= 5; *k *= 3). A *black *square represents the presence of a compound *j *(column index) in test *i *(row index). The *red *dashed line represents the separation of the STD design into its 3 layers (*k*), each of size 5 × 20 (*q *× *n*). Each compound is present *only once *in each layer of the design. Each compound is present *at most *once (Γ = 1) with every other compound.

A STD-based pool construction starts with the specification of the compound library size (*n*), maximum number of active compounds expected (*d*) and maximum number of errors expected (*E*). The STD algorithm *guarantees *that the pooled design will be able to correctly identify upto *d *active compounds in the presence of upto *E *false positive and negative errors in the screen. STD is able to provide such guarantees because it uses a combinatorial procedure to ensure that no two compounds are pooled together more than a minimum number of times, to prevent confounding decoding results. Also, the number of compounds pooled in each test is roughly the same, ensuring correct intensity-concentration mapping for the test results. This implies that for any underlying structure of compound activities and testing errors, as long as their numbers lie within the specified experimental parameters, the STD design *guarantees *successful identification of the active compounds. It has been observed [12] and recently shown [13] that pooling designs are capable of correcting errors much larger than those that they guarantee for. These input parameters (*n*, *d*, *E*) are used to choose the design parameters of the STD construction algorithm *q *and *k*. STD is a layered construction with *k *layers, each of size *q *× *n*. Each compounds appears only *once *in each layer. The STD construction algorithm produces a *t *× *n*, 0–1 matrix *M *= STD(*n*; *q*; *k*), with *t *(= *q *× *k*) rows that are the assays to be performed and *n *columns that represent the compounds in the library. The columns with entry 1 in a row are the compounds to be pooled in that assay. An example of such a pooling design is shown in Figure 1, created using *M *= STD(20; 5; 3) for *n *= 20, *d *= 2 and *E *= 0. The process of mapping the experimental parameters to the design parameters is shown below in Algorithm 1.

Algorithm 1:

Inputs – *n*, *d *and *E*

1. Choose a prime number *q*, with *q *<*n*. Start with the smallest prime, 2.

2. Find the *compression power*, Γ = min{*γ*|*q*^*γ*+1 ^≥ *n*}, therefore $\Gamma =\left\lceil \frac{\log n}{\log q}\right\rceil -1$. Set *k *= *d*Γ + 2*E *+ 1.

3. Check if this choice of *q *and *k *satisfy the *guarantee *requirements of identifying *d *active compounds and correcting *E *errors, using the inequality, *k *≤ *q *+ 1.

4. If the inequality is satisfied continue to step 5, else choose the next prime in step 1 and repeat steps 2 and 3.

5. Once the smallest prime number *q*_min _and its corresponding compression power Γ_max _are found, all *q *> *q*_min _will satisfy the inequality in step 3. Therefore, cycle through the values of Γ in {1, ..., Γ_max_} to find the corresponding *q*. For each Γ find the smallest *q *that satisfies, *q *≥ *n*^1/Γ +1^.

6. Calculate the number of tests (*t*) needed by each *q *and *k *pair from, *t *= *q *× *k*.

7. Choose the *q *and *k *pair producing the least number of tests.

8. Design the pooling matrix, *M *= STD(*n*; *q*; *k*).

A detailed description of the procedure used to construct the *t *× *n*, 0–1 matrix *M *can be found in the original paper [12] and is reproduced in the Methods section (Construction 1) of this paper.

Having designed the pooling scheme, *M*, the decoding algorithm is given below. The pooled assays, *t *in number, are carried out and the results classified into two states – positive or negative, using a chosen threshold.

1. A compound present in at least *E *+ 1 negative tests is tagged inactive.

2. A compound present in at least *E *+ 1 positive tests, in which all other compounds have been tagged inactive, is tagged active.

Note that each compound is present in *d*Γ + 2*E *+ 1 tests and no two compounds are mixed together more than Γ times. A more elaborate decoding algorithm designed to handle the presence of larger-than-designed-for values of *d *and *E *is provided in the original paper and its MATLAB implementation is provided [see Additional file 1].

### Error rate

**Figure 2:**
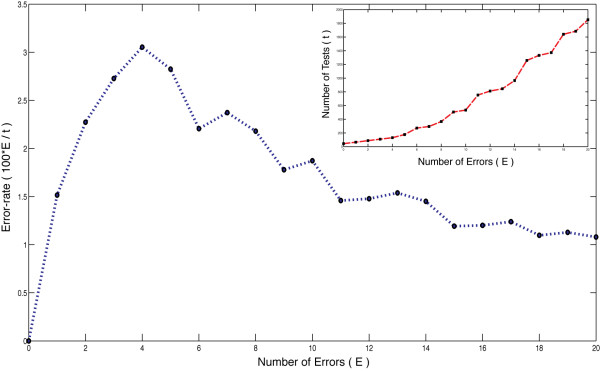
**Variation of error-rate**. Variation of error-rate ($e=\frac{E}{t}\times 100$) with choice of design parameter *E *(number of errors) keeping compound library size (*n *= 100) and expected number of positives (*d *= 3) fixed. The inset shows the variation in number of test needed (*t*) with the same parameter *E*, for a *E *= 0 to 20 and *t *= 44 to 1852.

The STD-based pooling strategy creates a design for a specified number of errors, *E*, resulting in the addition of 2*E *extra tests to the total of *t *pooled tests. Assuming random experimental errors, the number of errors present in the result will increase as the number of tests increase. In HTS, there can be instrument, biological, chemical, or human errors that increase (false positive, FP) or decrease (false negative, FN) the measurement from its true value [10]. These multiple sources of error make it difficult to estimate the total number of errors, *E*, before knowing the number of pooled tests (*t*) that will be used. However, often in HTS we know the overall random error-rate for an assay, which we call *e*. For example, the pooling design for 100 compounds (*n*) expecting 3 active compounds (*d*) and 2 testing errors (*E*) needs 88 tests (*t*) using STD(100; 11; 8) (Table 2 in [12]). If we define the error-rate (*e*) as the percentage errors expected per test then $e=\frac{E}{t}\times 100$. For the example above, this error-rate *e *is ~2.27%. However, when the number of errors (*E*) is changed, keeping everything else the same, the corresponding number of pooled tests (*t*) and hence the error-rate (*e*) change. From Figure 2 it can be seen, that the error-rate (*e*) reaches a maximum and then drops off with respect to *E*. The STD construction is efficient for low values of *E *but requires many more tests (*t*) for higher *E *values, as seen in Figure 2. This nonlinear relationship between *t *and *E *indicates that a construction based on the input parameter *E *alone is not satisfactory. We propose the use of an expected error-rate *e *as a input parameter instead. To do so we have modified the STD strategy as follows.

Algorithm 2: Error-rate based STD strategy

Inputs – *n*, *d *and *e*

Steps 1 and 2 same as (Algorithm 1)

3. If *d*Γ ≤ *q*, calculate $E_{\max}=\left\lfloor \frac{q-d\Gamma }{2}\right\rfloor$, the maximum number of errors that can be corrected by this choice of *q*, for the given *n *and *d*.

4. Check if the input error-rate *e *is achievable using the inequality, $e\leq \frac{E_{\max}}{q(d\Gamma +2E_{\max}+1)}\times 100$. If *e *is achievable then continue to step 5, else go back to step 1 and try the next *q*.

5. Cycle through all values for *E *from 0 to *E*_max _to find the minimum *E *(*E*_min_) that satisfies the inequality in step 4. Use *E*_min _to calculate, *k *= *d*Γ + 2*E*_min _+ 1 and the number of tests, *t *= *qk*.

6. Similar to Algorithm 1, cycle through the values of Γ smaller than the Γ_max _to find the corresponding *q *and hence *t*.

7. Use the design parameters (*q *and *k*) that need the minimum number of tests.

8. Construct the pooling design, *M *= STD(*n*; *q*; *k*), as usual.

¿From step 4 we find that there is an upper limit to the error-rate (*e*) for a given library size (*n*) and chosen maximum number of active compounds expected (*d*). This limit is a function only of the design parameter *q *as shown in Equation 1 (elaborated in Proof 1 of the Methods section).

(1) $$\begin{array}{cc} e\leq \frac{\left\lfloor \frac{q-d\Gamma (q,n)}{2}\right\rfloor }{q(d\Gamma (q,n)+2\left\lfloor \frac{q-d\Gamma (q,n)}{2}\right\rfloor +1)}\times 100, & \Gamma =\left\lceil \frac{\log n}{\log q}\right\rceil -1 \end{array}$$

**Figure 3:**
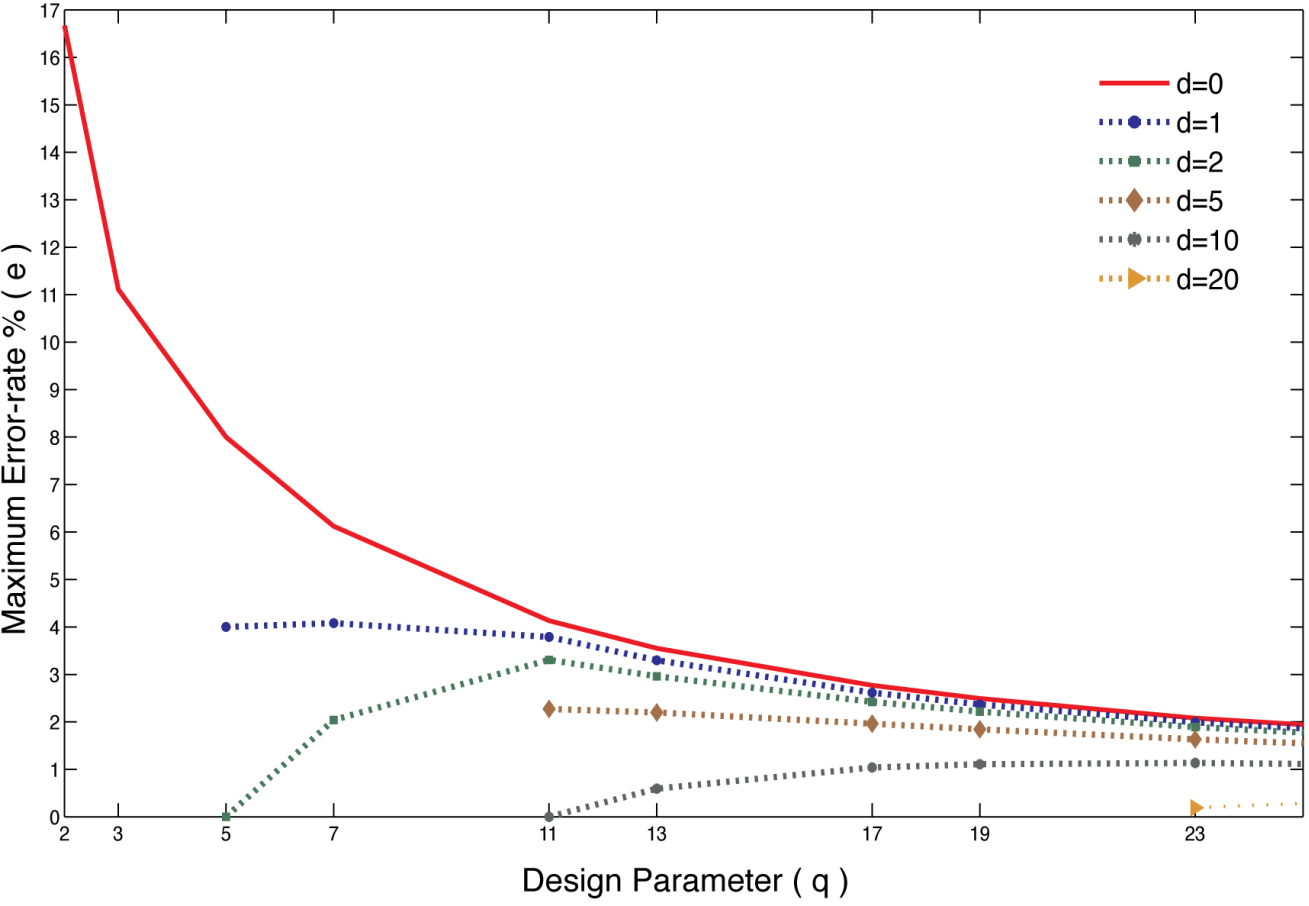
**Error-rate limit**. The limits on assay error-rate (*e*) that can be handled by STD for a sample choice of library size (*n *= 100) and various choices of expected active compounds (*d*). The trend suggests that the ability to correct for experimental assay error-rates decreases as *q *increases.

For the simplest case of *d *= 0, this expression simplifies to $e\leq \frac{q-1}{2q^{2}}\times 100$, for all the odd prime numbers and *e *≤ 16.67% for *q *= 2. Thus any assay with an error rate greater than 16.67% cannot *guarantee *accurate results when screened using an STD-based scheme. In more realistic cases, the error-rates corrected by STD-based schemes are much smaller than this limiting case of *d *= 0 because *d *and Γ would have substantial values. This finding implies that no matter how large the specified number of errors (*E*) in the original STD-based pooling strategy, the corresponding number of tests needed (*t*) would adjust itself to keep the error-rate (*e*) corrected by the design low. An example of the effect of Equation 1 can be seen in Figure 3 for a library of 100 compounds (*n *= 100) and various values of expected active compounds (*d*).

### Mixing Constraint

To our knowledge, no pooling designs address the physical limitation that only a finite number of compounds can be mixed in each well. This limitation arises in drug screening for the following three reasons.

1. Each compound must be present at a sufficiently high concentration so as to be detectable by the assay within a physiologically reasonable range.

2. The total ionic strength of the test solution must be low enough to prevent precipitation of compounds or possible changes to the biological target.

3. The assay must be reasonably simple to physically construct.

From these constraints we can conclude that the total number of drugs that can be practically included in any experimental test well is relatively small. For example, for a relevant screening concentration of ~10 *μ*M, we can assume that each well can have ~10 compounds mixed in it. If more drugs are mixed, the cost of creating the assay increases and the ionic strength of the well mixture may become too high, resulting in inaccurate screening results. This limit can be specified as an input parameter to the pooling design, based on the high-throughput assay being implemented.

Here we extend the STD-based pooling strategy to introduce an explicit mixing constraint. Let *m *be the mixing constraint, defined as the maximum number of compounds mixed in a well of the pooling scheme. Normally the STD design mixes at most $\left\lceil \frac{n}{q}\right\rceil$ compounds in each well [12]. However, this feature can break down when the input values of *n*, *d *and *e *(hence *E*_min_) are such that, for a certain choice of *q *and *k *(= *d*Γ + 2*E*_min _+ 1), *k *= *q *+ 1 and $\left\lceil \frac{n-1}{q^{\Gamma }}\right\rceil$ <*q *- 1, resulting in an unusually large number of compounds mixed in some tests and some tests with no compounds in them. The original STD construction can be easily modified by removing tests with no compounds in them. The details of the correction are provided in Proof 2 of the Methods section. Having made this correction, we can implement the mixing constraint as follows [see Additional file 2].

Algorithm 3: Mixing constraint implementation

Inputs – *n*, *d*, *e *and *m*

Steps 1 through 5 are the same as (Algorithm 2).

6. Using the *q *and *k *values obtained so far in the algorithm to choose one of the following options.

a. If *k *<*q *+ 1 and $\left\lceil \frac{n}{q}\right\rceil$ ≤ *m*, use Construction 1 (Methods section) requiring *t *= *qk *tests.

b. If *k *= *q *+ 1, *n *= *q*^Γ+1 ^- 1 and *q*^Γ ^≤ *m*, use Construction 1 requiring *t *= *q*(*q *+ 1) tests.

c. If *k *= *q *+ 1, $\left\lceil \frac{n-1}{q^{\Gamma }}\right\rceil$ <*q *- 1 and *q*^Γ ^≤ *m*, use Construction 2 (Methods section) requiring *t *= *q*^2 ^+ $\left\lceil \frac{n-1}{q^{\Gamma }}\right\rceil$ + 1 tests.

7. Similar to the previous algorithms, cycle through the values of Γ smaller than Γ_max _to find the corresponding *q *and hence *t*.

8 Use the design parameters (*q *and *k*) that require the minimum number of tests.

### Repeated Blocks

Limiting the number of compounds that can be mixed in a pooling strategy reduces the savings in tests that could otherwise have been obtained. For example, without the mixing constraint, screening a library of 10,000 compounds with 3 expected actives and no error requires only 110 tests, using STD(10000; 11; 10), each mixing 910 (=$\left\lceil \frac{10000}{11}\right\rceil$) compounds in them. However, a mixing constraint of 10 compounds per test increases the required tests to 4036, using STD(10000; 1009; 4). Now, consider the STD-based strategy for 400 compounds while expecting only 1 active compound in a similar error-free setting and a mixing constraint of 10 compounds per well. The number of tests needed in this case is 82, using the STD(400; 41; 2) design. If we now divide the original 10, 000 compound library into 25 blocks of 400 compounds each and used the STD(400; 41; 2) design repeated on those 25 blocks, the total number of tests is only 25 × 82 = 2050, almost half the original requirement! The trade-off here is that the block assay design guarantees the detection of only 1 active compound out of every 400 compounds tested. However, given that we expected only 3 active compounds in a library of 10, 000 compounds, this implies that there is ~99.5% chance of finding, at best, 1 active compound among the 400 randomly selected compounds for a block (using the hypergeometric distribution). This block decomposition algorithm is inspired by a form of pooling used in NMR screening [14]. This example demonstrates that we can make an informed choice about a block size that can, not only, help reduce the number of tests needed while enforcing the mixing constraint but the smaller block size also implies that a better error-rate can be handled by the design (as seen in Figure 3).

For any block of *n*_*B *_compounds from the library of *n *compounds, the number of blocks needed to cover the whole library is $B=\left\lceil \frac{n}{n_{B}}\right\rceil$. The creation of a STD for this block of *n*_*B *_compounds, using the error-rate *e *and mixing constraint *m *(assumed to stay the same for all blocks) requires the specification of the maximum number of active compounds expected for the given *n*_*B*_, called *d*_*B*_. The choice of *d*_*B *_is from a hypergeometric distribution such that, with at least a probability of *p*_*B *_(specified at the outset) the block of *n*_*B *_compounds contains at most *d*_*B *_active compounds. Using the hypergeometric distribution, the following inequality (elaborated in Proof 3 in the Methods section) can be solved for *d*_*B*_.

(2) $$\sum_{i=0}^{d_{B}}\frac{\left(\begin{array}{c} d\\ i \end{array}\right)\left(\begin{array}{c} n-d\\ n_{B}-i \end{array}\right)}{\left(\begin{array}{c} n\\ n_{B} \end{array}\right)}\geq p_{B}$$

Based on this choice of *d*_*B*_, a STD-based pooling design of size *t*_*B *_× *n*_*B *_can be generated using Algorithm 3. The number of tests needed for the whole library would be *B *× *t*_*B*_.

### poolHiTS

We now define poolHiTS as an STD-based pooling strategy which takes in as input the compound library size (*n*), maximum number of active compounds expected (*d*), maximum error-rate expected (*e*), mixing constraint (*m*), and design confidence metric (*p*_*b*_). poolHiTS(*n*, *d*, *e*, *m*, *p*_*b*_) produces a *t *× *n *mixing matrix *M*, which guarantees the success of the pooling scheme for the given input parameters. The algorithm for poolHiTS, which includes error-rate specification, mixing constraints, and optimal block size selection, is as follows. The MATLAB implementation of the poolHiTS algorithm is provided [see Additional file 3].

Algorithm 4: poolHiTS algorithm

Inputs – *n*, *d*, *e*, *m *and *p*_*B*_

1. Choose a value of *d*_*B *_in {1, ...,*d *- 1}.

2. Find the set of *n*_*B *_that satisfy the inequality in Equation 2.

3. For each *n*_*B *_in this set use Algorithm 3 to evaluate STD(*n*_*B*_; *q*_*B*_; *k*_*B*_), if it exists, for the given *e *and *m*. Calculate the total number of tests needed from *B *× *t*_*B*_, $B=\left\lceil \frac{n}{n_{B}}\right\rceil$.

4. Choose the next value of *d*_*B *_and repeat steps 2 and 3.

5. After testing all values of *d*_*B *_in {1, ...,*d *- 1} and the corresponding *n*_*B*_, select the *d*_*B*_, *n*_*B *_pair that require the least number of tests. The whole library design (which corresponds to *n*_*B *_= *n*, *d*_*B *_= *d *and *B *= 1) should also be included while making this choice.

6. Design the pooling matrix, *M *= STD(*n*; *q*; *k*), for the choice of *q *and *k*.

A typical example of a result of applying Algorithm 4 to the pooling design problem is shown in Table 1. Consider a case of a 10, 000 compound library (*n*), where we expect upto 3 active compounds (*d*) with 1% assay error-rate (*e*), a limit on mixing not more than 10 compounds in a test (*m*), and at least a 99% chance of finding the active compounds (*p*_*B*_). From this problem specification we have two observations. First, a single whole library design is not possible because the mixing constraint permits only 10 compounds per assay, thus we must use a repeated block design. Second, we see that there is an optimal number of blocks (*B*) that requires the least number of tests and has a block size (*n*_*B*_) between the two extremes (*n*_*B *_= *n *and *n*_*B *_= 1). The best design chosen by poolHiTS(10000, 3, 1, 10) is to implement a block pooling scheme for *n*_*B *_= 110, *d*_*B *_= 1, *e *= 1 and *m *= 10, using STD(10000; 11; 4), and repeat this design 91 times over to cover the whole library. The reason for choosing *n*_*B *_= 110 rather than *n*_*B *_= 130 (see Table 1), while both provide equal compression, is that the former provides a better actual error-correcting rate of 2.27%. It is useful to note that this design is capable of screening a library of 91 × 110 = 10,010 compounds vs. the specified 10, 000 compounds. However, because of the extra error-correction, we can take advantage of this design by ignoring the extra compounds or using some form of control compound in their place. The MATLAB implementation of this example is provided [see Additional file 4].

**Table 1:** Block Design Example

Number of blocks (*B*)	Number of compounds per block (*n*_*B*_)	Number of active compounds expected per block (*d*_*B*_)	Number of tests needed per block (*t*_*B*_)	Actual error-rate handled (*e*)	Actual mixing constraint (*m*)	Total number of tests needed (*t*)
1	10000	3	-	-	-	-
25	400	1	492	1.02%	10	12300
50	200	1	92	1.09%	9	4600
**77**	**130**	**1**	**52**	**1.92%**	**10**	**4004**
**91**	**110**	**1**	**44**	**2.27%**	**10**	**4004**
100	100	1	44	2.27%	10	4400
500	20	1	20	5%	4	10000

Sample case of *n *= 10, 000, *d *= 3, *e *= 1% and *m *= 10 showing the efficiency of selected block design parameters. The choice of error-rate (*e *= 1%) does not permit a full library design (first row), thus it is left empty.

## Conclusion

In this paper, we present a pooling strategy called poolHiTS which implements tailored modifications and enhancements that make the shifted transversal design (STD) algorithm appropriate for drug discovery. First, we demonstrate how to switch from specifying the number of errors (*E*) for a STD-based strategy to an error rate (*e*), which is the percentage of errors expected in tests. We show that there is an upper limit to the error-rate that can be handled by this STD-based algorithm and this error limit (Equation 1) strongly constrains pooling designs to less noisy screening assays. We implement error rate as an input experimental parameter via Algorithm 2.

Second, we introduce and implement explicit mixing constraints to make pooling significantly more relevant to HTS assays via Algorithm 3. We also provide a necessary correction to the STD construction algorithm pertinent to the mixing constraint.

Third, we introduce the concept of repeated block designs that retains the efficiency of pooling strategies in the face of the mixing constraint and error-rate limitations. We show that by using this block design, we are able to simplify the assay construction, increase the error tolerance and decrease the assay size. The combination of these features produce the poolHiTS strategy described in Algorithm 4. The MATLAB implementations of the poolHiTS algorithm and an example of its use are provided in the Additional Files section. poolHiTS provides a promising route to both reducing the cost and increasing the accuracy of high throughput drug screening. Although poolHiTS primarily focuses on drug screening, these same design methods can apply equally well to other screening environments where the number of perturbations to a system is finite or small (mixing constraint) and the error rate of the assay is approximately known.

## Methods

### Construction 1

Given the design parameters *q *and *k*, the pooling matrix *M *= STD(*n*; *q*; *k*) can be constructed as follows. The STD has a layered construction consisting of *k *layers of *q *× *n *boolean matrices. For all *j *∈ {0, ..., *k *- 1}, let *M*_*j *_be a *q *× *n *boolean matrix representing layer *L*(*j*), with columns *C*_*j*,0_, ...,*C*_*j*,*n*-1_.

Let the circular shift operator, *σ*_*q*_, be defined as, ∀(*x*_1_, ..., *x*_*q*_) ∈ {0,1}^*q*^, $\sigma_{q}\left[\begin{array}{c} x_{1}\\ x_{2}\\ \vdots \\ x_{q} \end{array}\right]=\left[\begin{array}{c} x_{q}\\ x_{1}\\ \vdots \\ x_{q-1} \end{array}\right]$ and $C_{0,0}=\left[\begin{array}{c} 1\\ 0\\ \vdots \\ 0 \end{array}\right]$. Note that *σ*_*q *_is a cyclic function and when applied *q *times maps {0, 1}^*q *^onto itself, $\sigma_{q}^{s}\left[\begin{array}{c} x_{1}\\ x_{2}\\ \vdots \\ x_{q} \end{array}\right]=\left[\begin{array}{c} x_{1}\\ x_{2}\\ \vdots \\ x_{q} \end{array}\right]$, *s *= *q*. To design a layer *L*(*j*), for all *i *∈ {0, ..., *n *- 1} construct $C_{j,i}=\sigma_{q}^{s(i,j)}C_{0,0}$ where,

• if *j *<*q*: $s(i,j)=\sum_{c=0}^{\Gamma }j^{c}\left\lfloor \frac{i}{q^{c}}\right\rfloor$

• if *j *= *q*: $s(i,q)=\left\lfloor \frac{i}{q^{\Gamma }}\right\rfloor$

The layers *L*(*j*) are put together to form *M *by, $\text{STD}(n;q;k)=\overset{k-1}{\underset{j=0}{\cup }}L(j)$. A detailed description of the construction is available in the original STD paper [12].

### Proof 1: Error-rate limit

Given *n*, *d *and *E*, the STD algorithm uses a prime number *q *(<*n*) and a compression factor $\Gamma (=\left\lceil \frac{\log n}{\log q}\right\rceil -1)$ which satisfy the inequality, *d*Γ + 2E ≤ *q*. The number of tests required for the design is, *t *= *q*(*d*Γ + 2*E *+ 1). The error-rate corrected by this design can be defined as, $e=\frac{E}{t}\times 100$. However, for a choice of *q *that satisfies the design requirement there is a upper limit on the number of errors the corresponding design can correct, given by, *E*_max _= $\left\lfloor \frac{q-d\Gamma }{2}\right\rfloor$ (arrived at from the inequality above). This implies that the error-rate (*e*) that can be specified for a design is constrained in the following manner.

(3) $$e\leq \frac{E_{\max}}{t}\times 100$$

Using the expression for *t*,

(4) $$e\leq \frac{E_{\max}}{q(d\Gamma +2E_{\max}+1)}\times 100$$

Substituting for *E*_max_,

(5) $$e\leq \frac{\left\lfloor \frac{q-d\Gamma }{2}\right\rfloor }{q(d\Gamma +2\left\lfloor \frac{q-d\Gamma }{2}\right\rfloor +1)}\times 100$$

Using the above inequality it is possible to deduce the maximum error-rate that STD can handle for a particular *n*-compound library with at most *d *active compounds and an assay with a limit on mixing not more than *m *compounds in a test. However, in most cases, the numerical value of the maximum error rate can only be calculated by executing (Algorithm 3) completely. For the simplest case of *d *= 0, since *q *is a prime number and hence odd (except for *q *= 2), the term $\left\lfloor \frac{q-d\Gamma }{2}\right\rfloor$ is reduced to $\left\lfloor \frac{q}{2}\right\rfloor$ and is always equal to $\frac{q-1}{2}$. Simplifying the expression for the error limit, we get, $e\leq \frac{q-1}{2q^{2}}\times 100$. Whereas, for *q *= 2 (the only even prime number), the value of the limit is, *e *≤ $\frac{1}{6}$ × 100 ~ 16.67%. This is the maximum error-rate that STD-based designs can guarantee correction for.

### Proof 2: STD Correction

In the case where the choice of the design parameters *q *and *k *(= *d*Γ + 2*E*_min _+ 1) are such that, *k *= *q *+ 1, then the construction of the last layer (*j *= *k *- 1, *k *= *q *+ 1) will use *s*(*i*, *q*) = $\left\lfloor \frac{i}{q^{\Gamma }}\right\rfloor$ (see *Construction *1).

Since each layer *L*(*j*) consists of *q *rows and *n *columns and these are constructed by applying the circular shift operator (*σ*_*q*_), *s*(*i*, *q*) times, on *C*_0,0 _where *i *∈ {0, ..., *n *- 1}. By definition *q*^Γ ^<*n *≤ *q*^Γ+1^, *s*(*i, q*) takes the values {0, 1, ..., *q *- 1} sequentially. As shown in the illustration below, for columns *i *∈ {0, ..., *q*^Γ ^- 1}, *s*(*i*, *q*) = 0 and hence they all resemble *C*_0,0 _exactly. The next *q*^Γ ^columns have *s*(*i*, *q*) = 1 and resemble *C*_0,0 _once shifted and so on.

$$\begin{array}{cccccccccccccccc} \text{row}/\text{col}. & 0 & \ldots & q^{\Gamma }-1 & q^{\Gamma } & \ldots & 2q^{\Gamma }-1 & \ldots & \ldots & \ldots & n-1 & & \ldots & (q-1)q^{\Gamma } & \ldots & q^{\Gamma +1}-1\\ 0 & 1 & 1 & 1 & 0 & 0 & 0 & 0 & 0 & 0 & 0 & & \ldots & 0 & 0 & 0\\ & 0 & 0 & 0 & 1 & 1 & 1 & 0 & 0 & 0 & 0 & & \ldots & 0 & 0 & 0\\ \vdots & & \vdots & & & & \vdots & & & & & & & & \vdots & \\ \left\lceil \frac{n-1}{q^{\Gamma }}\right\rceil & 0 & 0 & 0 & 0 & 0 & 0 & 0 & 1 & 1 & 1 & & \ldots & 0 & 0 & 0\\ & 0 & 0 & 0 & 0 & 0 & 0 & 0 & 0 & 0 & 0 & & \ldots & 0 & 0 & 0\\ \vdots & & \vdots & & & & \vdots & & & & & & & & \vdots & \\ q-1 & 0 & 0 & 0 & 0 & 0 & 0 & 0 & 0 & 0 & 0 & {\;}_{q\times n} & \ldots & 1 & 1 & 1 \end{array}$$

When the library size *n *is such that $\left\lceil \frac{n-1}{q^{\Gamma }}\right\rceil$, only rows from 0 to $\left\lceil \frac{n-1}{q^{\Gamma }}\right\rceil$ have compounds in them and the rest of the rows in the layer are empty. It follows that in this layer the number of compounds mixed in each test is *q*^Γ^. Usually, the number of compounds per test is $\left\lceil \frac{n}{q}\right\rceil$ and in this case we know that $\frac{n}{q}<q^{\Gamma }$. Hence, it is essential to make this correction in order to correctly implement the mixing constraint. For example, In Table 2 of the original STD paper [12], there is a case where *n *= 10000, *d *= 3 and *E *= 2 with the optimal choice of *q *= 13 and *k *= 14. In this case *k *= *q *+ 1, so the actual number of tests is *t *= 174, and not 182 (= 13 × 14) as stated in the paper, and the maximum number of compounds being mixed is actually 2197 rather than 769.

### Construction 2

A modification is required for the special case where the choice of design parameters (*q *and *k*) is such that *k *= *q *+ 1 and $\left\lceil \frac{n-1}{q^{\Gamma }}\right\rceil$ <*q *- 1. The construction remains essentially the same as Construction 1 with the only modification being the removal of the unnecessary tests with no compounds in them. As shown in Proof 2, the last *q *- ($\left\lceil \frac{n-1}{q^{\Gamma }}\right\rceil$ + 1) rows of *M *need to be removed. It should be noted that the maximum number of compounds being mixed in a test is *q*^Γ^. The MATLAB implementation of this construction is provided [see Additional file 5].

### Proof 3: Choice of Block Size

The problem of block size selection is one of *sampling without replacement*. The compound library (*n*) with at most *d *active compounds has to be divided into *B *blocks of size *n*_*B *_and the probability of dividing these active compounds into different blocks needs to be determined. The hypergeometric distribution provides a way to estimate this probability. Thus, the probability of finding *i *active compounds in a block of size *n*_*B *_selected without replacement from the compound library of size *n *that contains *d *active compounds is given as,

(6) $$P(i)=\frac{\left(\begin{array}{c} d\\ i \end{array}\right)\left(\begin{array}{c} n-d\\ n_{B}-i \end{array}\right)}{\left(\begin{array}{c} n\\ n_{B} \end{array}\right)}$$

The identification of the experiment design parameter *d*_*B *_for the block requires the specification of a probability (*p*_*B*_) representing a *confidence *in finding at most *d*_*B *_active compounds in a block of size *n*_*B*_, which is the sum of all the individual probabilities of finding exactly *i *= {0, 1, ..., *d*_*B*_} active compounds out of *n*_*B *_compounds. Therefore the condition for calculating *d*_*B *_is $\sum_{i=0}^{d_{B}}P(i)\geq p_{B}$, thus arriving at Equation 2. It is useful to note that other design parameters of error-rate (*e*) and mixing constraint (*m*) are so defined, as to be the same for individual blocks and the whole library. The MATLAB implementation of this equation is provided [see Additional file 6].

## Authors' contributions

PJW framed the original problem, participated in algorithm development and helped draft the manuscipt. RMK developed the algorithms, implemented the software and drafted the manuscript. Both authors read and approved the final manuscript.

## Supplementary Material

Additional file 1 The MATLAB code used to implement the poolHiTS algorithm is provided. It includes the advances described in this paper, such as, the error-rate, mixing constraint and the block design strategy. This subroutine implements the standard STD decoding algorithm to identify the active compounds using the result *T *of a pooled experiment, *M*. Click here for file

Additional file 2 The MATLAB code used to implement the poolHiTS algorithm is provided. It includes the advances described in this paper, such as, the error-rate, mixing constraint and the block design strategy. This subroutine executes Algorithm 3 for a given set of parameters *n*, *d*, *e*, *m *to obtain the optimal values of *q *and *k*. Click here for file

Additional file 3 The MATLAB code used to implement the poolHiTS algorithm is provided. It includes the advances described in this paper, such as, the error-rate, mixing constraint and the block design strategy. This subroutine executes Algorithm 4 for a given set of parameters *n*, *d*, *e*, *m*, *p*_*B *_to choose the optimal block configuration of *B *and *n*_*B*_. It calls the poolHiTS-design.m and poolHiTS-hypgeo.m subroutines. Click here for file

Additional file 4 The MATLAB code used to implement the poolHiTS algorithm is provided. It includes the advances described in this paper, such as, the error-rate, mixing constraint and the block design strategy. This is the main file that needs to be executed, where the parameters *n*, *d*, *e*, *m *can be set and in-silico experiments can be run. It utilizes the poolHiTS.m subroutine for choosing the design, the poolHiTS-const.m subroutine for construction and the poolHiTS-decode.m subroutine for decoding the results of a pooled assay. Click here for file

Additional file 5 The MATLAB code used to implement the poolHiTS algorithm is provided. It includes the advances described in this paper, such as, the error-rate, mixing constraint and the block design strategy. This subroutine executes Construction 2 for a given set of parameters *n*, *q *and *k*. Click here for file

Additional file 6 The MATLAB code used to implement the poolHiTS algorithm is provided. It includes the advances described in this paper, such as, the error-rate, mixing constraint and the block design strategy. This subroutine evaluates the left hand side of Equation 2 for a given set of *n*, *d*, *n*_*B *_and *d*_*B*_. Click here for file
